# Older patients’ participation in hospital admissions through the emergency department: an interview study of healthcare professionals

**DOI:** 10.1186/s12913-015-1136-1

**Published:** 2015-10-21

**Authors:** Dagrunn Nåden Dyrstad, Ingelin Testad, Marianne Storm

**Affiliations:** Department of Health Studies, Faculty of Social Sciences, University of Stavanger, Stavanger, Norway; Department of Anaesthesiology and Intensive Care, Stavanger University Hospital, Stavanger, Norway; Centre for Age-Related Medicine, SESAM, Stavanger University Hospital, Stavanger, Norway

**Keywords:** Patient participation, Views of healthcare professionals, Interviews, Hospital admission, Older patients

## Abstract

**Background:**

Patient participation is an important aspect of healthcare quality and may be one way to improve the quality of transitional care for older patients. Research reveals minimal awareness about patient participation in hospital admissions. Hospital admissions require attention to individuals’ specific needs beyond patient frailty, and to involve patients and their families in shared decision-making. The aim of this study was to identify factors influencing patient participation by exploring healthcare professionals’ views on patient participation during the hospital admission of older patients through the emergency department (ED).

**Methods:**

The study used a qualitative and descriptive design with face-to-face interviews. A total of 27 interviews were conducted with 15 healthcare professionals from one hospital and 12 from another. The data were analyzed using systematic text condensation.

**Results:**

Healthcare professionals thought that patient participation in hospital admissions was influenced by five main factors: 1) routine treatment and care during hospital admission, and in particular certain procedures such as medical examinations; 2) the frail and thankful older patients, and the overall picture of their medical needs; 3) hospital resources, such as available staff and beds; 4) healthcare professionals’ attitude towards finding out about older patients’ experiences; and 5) the presence of a supportive and demanding next of kin acting as an advocate for the patient.

**Conclusions:**

Patient participation in hospital admissions of older patients is dependent on the way the service is organized, the patients’ condition, hospital resources, healthcare professionals’ attitudes, and support from patients’ next of kin. Some of the participants had high expectations of themselves and actively involved patients, but others did not find patient participation relevant in the emergency department. Some used crowded wards as a reason not to engage older patients in their own care.

## Background

Patient participation is one way to improve healthcare quality [1–3]. It can be viewed as a response to the paternalistic healthcare model, in which the patient has a passive, dependent role and the physician or healthcare professional is the expert on treatment and care [4–6]. Patient participation includes the patient’s right to participate in decision-making about treatment and care, level of care, and living conditions [7]. During hospital admissions, providing information to patients about planned tests and treatment, and the planned stay in hospital, and giving them opportunities to describe their symptoms (what has happened and how) are important to ensure patient involvement [8]. Transitional care, which includes hospital admission, was defined by Coleman and Bolt [9] as a set of actions to ensure the quality and continuity of healthcare as patients transfer between hospital and community healthcare services [10].

Older people with multiple diseases and medication have complex care needs and often transfer between community and hospital healthcare services [11–13]. These patients are a vulnerable group at the point of hospital admission and may have difficulties self-advocating owing to illness, confusion, or deterioration of health [13]. Research shows that older patients in the emergency department (ED) often do not remember whether they have received information about treatment or been involved in decision-making about treatment and hospital admission [8, 14, 15]. Patients who are admitted as an emergency also report receiving less information about the results of their medical treatment and care [16]. A lack of information exchange between healthcare professionals and gaps in the documentation about patients’ cognitive function, mental orientation, medication charts, and advance directive status can also complicate the transfer of older patients to the ED [13]. Such gaps will require healthcare professionals to spend more time to ensure that adequate and individualized care is provided to the patient in the ED.

Studies report that healthcare professionals do not always focus on patient participation. Some are aware of involving patients in decisions concerning their treatment and care, while others lack competencies in this area [15, 17–21]. In particular, at the point of hospital admission, with time pressure and a strong emphasis on efficiency, clinicians can easily focus on medical problems and not patients’ individual preferences and opinions [19, 20, 22].

It can be challenging for healthcare professionals to look beyond the frailty, complex medical history and multiple medications of older people in the ED, and instead focus on the individual’s preferences and views [23]. A common and important screening tool used by healthcare professionals in the ED is the emergency severity index triage system, which scores patients from 1 (most urgent) to 5 (least resource-intensive) [24–26]. The triage system provides timely clinical observations, tests, and examinations to support decisions about treatment and care. It does not, however, automatically include patient involvement in decision-making and can result in failure to see the patient as a whole person. The aim of our study was to identify factors influencing patient participation by exploring healthcare professionals’ views on patient participation during the hospital admission of older patients through the ED.

## Methods

### Design

The study applied a qualitative and descriptive design. The descriptive approach is rooted in Giorgi’s phenomenological research, which focuses on individual experiences in their natural context [27]. A descriptive design aims to provide an “accurate portrayal of the characteristics of persons, situations, or groups and/or the frequency with which certain phenomena occur” [28]. We conducted face-to-face individual interviews to gather descriptions of the diversity and nuances in healthcare professionals’ views on patient participation, to increase understanding of this complex phenomenon [29].

### Participants and study setting

We held individual interviews with ambulance workers, nurses, and doctors in two hospitals in the same Regional Health Authority in Norway, one hospital with 595 patient beds and one hospital with 206 patient beds. The reason for choosing two hospitals was to explore different contexts [30].

All the participating nurses worked in the ED (triage unit and treatment rooms), providing nursing care for incoming patients. The ambulance workers were from the ambulance station connected to the hospital. Their work tasks included responding to emergency calls, transporting patients to the hospital, and triaging patients based on the severity of their illness. The medical doctors in the study were based in either medical or orthopedic hospital wards, serving the ED in their specialist area. The interns had a schedule that rotated between medical and orthopedic wards while they were working in the ED.

### Data collection

The leader of each of the three professional groups (ambulance workers, nurses, and doctors) gave approval for the interviews to be conducted with staff members. A total of 29 healthcare professionals were invited to participate in individual interviews during work hours between March and October 2012, and 27 agreed to do so. The remaining two cited high workloads and change of work schedule as their reasons for not participating. The interviews with nurses and interns took place in an office in the ED, ambulance workers were interviewed in an office at the ambulance station, and medical doctors were interviewed in their own offices. Table 1 shows information about the participants.

**Table 1:** Interviews with hospital healthcare professionals

Profession	Gender, age	Professional work experience in field
8 ambulance workers	2 females, 6 males	Mean 15 years
	Mean age 41	
9 nurses	9 females	Mean 8 years
	Mean age 46	
4 medical doctors	1 female, 3 males	Mean 5 years
	Mean age 36	
6 interns	4 females, 2 males	Mean 6 weeks
	Mean age 28	

A semi-structured interview guide was developed based on the study protocol of the main study [30] and four previous studies [10, 19, 31, 32]. This included the following main topics: (1) coordination/interaction among care providers (experiences, success, problems, and improvements), (2) multidisciplinary collaboration, (3) information exchange, (4) knowledge sharing, (5) quality and safety, (6) patient and family involvement/education, (7) structure/planning, and (8) challenges/barriers. Each interview lasted approximately 1 h and was audio-taped.

### Ethical considerations

The Western Norway Regional Ethics Committee for Medical Research approved the study (REC, no.2011/1978). Participation was based on informed oral and written consent. The interview participants received an information letter from their professional lead describing the project’s aims and focus. The researcher contacted the healthcare professionals after they had been informed about the study and agreed to participate.

### Data analysis

The audiotaped interview data material was transcribed to text format (274 pages) by a professional editor and by the first author of this article (half each). The first author then read all of the text transcripts to validate the written interview data. The interview data were analyzed using a systematic text condensation approach [33]. To ensure trustworthiness in the analysis, the three authors met to discuss, analyze, and code the interview data [28]. The researchers performed a four-step analysis, in part together and partly individually, before and after meeting:

1. The authors separately read the data material several times to obtain an overall impression before they met, and each presented their preliminary themes at the meeting.
2. Meaning units, or “text fragment[s] containing some information about the research question” (p. 797) [33]—in this case patient participation in hospital admission—were identified by all three authors beforehand and agreed upon during the meeting [33].
3. After the meeting, the first author continued to work on identifying meaning units related to the agreed themes. The meaning units were coded into code groups, which were sorted into subgroups by the first author, reducing and condensing the data but maintaining the original terminology as much as possible.
4. Finally, descriptions and concepts were discussed, and five categories were agreed upon [33].

Table 2 illustrates how the analysis proceeded using a selection of “meaning units” from the interview transcripts.

**Table 2:** Extracts from the interview analysis process

Preliminary themes	Codes/meaning units and code groups	Subgroups	Categories
Prerequisites for patient participation	Patient treatment and care	Necessary treatment of the patient	Routine treatment and care during hospital admission
	We observe the patient’s vital functions to provide correct treatment and care. (ambulance worker)	Take care of vital functions	
	We are not forcing the patients, but we have to do our procedures and routines; undressing the patients, getting them into hospital clothing, performing the medical examination, establishing a diagnosis and then we ask the patients if they have any questions (ED nurse)		
	Information	Information to and from the patient	
	Informing the patient is important so that he understands the medical problem and agrees to the planned treatment (intern)		
	Competence	Variable competence	
	Interns are unexperienced and need supervision (ED nurse)		
Barriers to patient participation	Older patients’ health status	Frail health status	The frail and thankful older patient
	Small changes in the older patients’ health condition lead to severe consequences (ambulance worker)		
	The challenge with older patients is the compound medical picture (medical doctor)	A compound medical picture	
	Belonging to another generation		
	Older patients never complain and tolerate pain very well, they do not want to bother anyone (medical doctor)	Older patients are thankful	
Barriers to patient participation	The time aspect	Time is limited	Hospital resources; available staff and beds
	We have limited time for the patients, so when older patients want to explain what is wrong, we sometimes have to stop them (medical doctor)	High workload	
	One has to prioritize, if you spend much time on one of the older patients, then there is less time for other patients in the ED (intern)	Priority of time	
How to conduct older patients’ participation	Respect	Involving the patient in practice	Healthcare professionals’ attitudes towards exploring older patients’ experiences
	I like working together with the patient (medical doctor)		
	I think it is of high importance that we show we care (ambulance worker)	Show that we care	
	The patients say what they want if you sit down and ask them (medical doctor)		
	I think it is important that the patient feel he has a right to decide himself and [to feel] that we do not just overrule him by our procedures, which we easily can (medical ED nurse)	Older patients want to stay at home	
	Preference for participation	Patient involvement in ED not relevant	
	Older people want to stay at home as long as possible if they know help will come when needed (ED nurse)	Multiple transitions	
	I don’t think patient participation is very relevant in the ED (ED nurse)		
	It is important to not treat the older patient as a packet and transfer him from place to place (ambulance worker)		
How to conduct older patients’ participation	Next of kin	The next of kin role	Presence of a supportive and demanding next of kin
	Older people often call the next of kin instead of the doctor or the emergency services (ambulance worker)	Next of kin is first priority	
	It is not easy to get any information from the older patient in a bad health condition; then next of kin supports with useful and necessary information (intern)	Next of kin, an information source	
	Patients are more heard if the next of kin is present in the admission situation (ED nurse)	Patients heard if next of kin present	
	Next of kin can be challenging, having their own interests, which are not always the same as the patient’s (medical doctor)	Next of kin’s interests unlike the patient’s	
	For a nurse it is good to know that the patient is not alone in the room, he has his family present, especially when I am busy with other patients. Then I ask them to tell me when they are leaving (ED nurse)	Family, safety for patient and nurse	

## Results

**Fig. 1:**
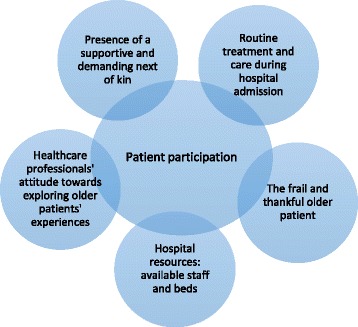
Primary factors influencing healthcare professionals’ views on older patients’ participation

Healthcare professionals’ views on patient participation during hospital admission of older patients were influenced by five factors, shown in Fig. 1:

1. Routine treatment and care during hospital admission;
2. The frail and thankful older patient;
3. Hospital resources: available staff and beds;
4. Healthcare professionals’ attitude towards exploring older patients’ experiences; and
5. Presence of a supportive and demanding next of kin

### Routine treatment and care during hospital admission

The first priority in the treatment and care of older patients on hospital admission is, according to the study participants, to save the patient’s life. The participants stated that all patients are triaged when they arrive in the ED, based on the severity of their illness. According to several ambulance workers, observations of the patients’ vital functions were necessary to provide correct and effective treatment and care both during the ambulance journey and in the ED. In the ED, one intern reported that medical examinations involve checking the patient’s physical functions using a top-to-toe checklist. One nurse suggested that leaving patients in bed can easily lead to them feeling vulnerable. She said:

“*We are not forcing the patients, but we have to do our procedures and routines; …undressing the patients, getting them into hospital clothing, performing the medical examination, establishing a diagnosis, and then we ask the patients if they have any questions.*”

Several of the interview participants, particularly interns and nurses, talked about how and why they provided information to their patients. They considered information was necessary for patients to understand their medical problem and agree to the planned treatment, and for them to feel safe and well cared-for. The amount and content of the information provided to patients varied, depending on the interviewee’s profession and whether the patient was in the ambulance or the ED. Ambulance workers said that they told the patient how long it would take to reach the hospital and explained the care that would be provided during the journey and at the hospital. They asked questions about the patients’ symptoms and current health condition so that they could meet patient needs. At the hospital, patients were often given minimal information by nurses in the triage part of the ED, because the patients were waiting for a medical examination. In the treatment part of the ED, the doctors (interns) usually provided information, often repeated by the nurses. Information focused on the possibilities and risks of surgery, medical treatment, and plans.

Views on how to involve patients to secure optimal treatment and care varied between the interview participants. One nurse held that a challenge to patient involvement was that the interns needed to focus on patients’ basic medical treatment before prioritizing their involvement. The interns held that they were inexperienced and needed supervision around procedures and medical examination from medical doctors, who were often not present in the ED.

### The frail and thankful older patient

Ambulance workers, nurses, and doctors all commented that the older patients in the ED were often frail, and had several chronic diseases and different sets of medication. They were therefore a challenging patient group to involve in their own treatment and care. According to the interview participants, these patients were often in need of help in many areas because of their hearing difficulties, trouble walking, or spells of dizziness and cognitive impairment. One medical doctor commented that the deterioration in the medical condition that had resulted in the hospital visit could exacerbate existing problems, and that older patients could often be very confused when admitted to the hospital.

The medical examination of frail older patients was described by participants as “complex”. One medical doctor commented that patients may have an acute medical problem combined with other conditions, which can make it difficult to find the medical reason for the problem on admission. Older patients were also characterized by one nurse as grateful for help. A medical orthopedic doctor agreed that they may seek help too late, and tend not to ask questions, but wait patiently to be seen by the doctor or nurse. This means that they are not involved in decisions on treatment and care. Participants felt that combination of a complex medical picture and the tendency to accept, and not complain, can lead older patients to be assessed as having simpler care needs than is actually the case.

Some nurses and doctors emphasized the importance of hearing from the patient. Some doctors said that they asked the patients to explain their health challenges and current problems during the medical examination:

*“The older patient has important information that is not documented by healthcare professionals, but the patient is at risk of not being heard. Sometimes there is a difference between the content of the written medical information from healthcare professionals in the municipality and what the patient says.”* (intern)

### Hospital resources: available staff and beds

Findings suggest that having sufficient staff and beds available constituted a challenge to patient participation for both hospital and municipality healthcare services. Several ambulance workers and an intern said that during the nights and weekends, staffing in nursing homes and home healthcare services is reduced. In their view, this could lead to patients being admitted to the hospital without adequate information about their medical history or medication.

A medical doctor said that this shortage of staff in the municipality meant that patients often had to be admitted to the hospital, rather than sent home. The number of available staff at the hospital, however, was also said to be lower during weekends and nights. One nurse suggested that the combination of an over-crowded ED and hospital wards, and the lack of staff could influence staff capacity to care for patients. Mondays were often busy because patients waited until after the weekend to contact the doctor.

The participants reported that obtaining a medical history from older patients could be time-consuming. One intern said that this could be because of complex medical status, multiple types of medication, and older patients’ difficulties explaining their health problems.

“*We have limited time for the patients, so when the older patients want to explain what is wrong, we sometimes have to stop them.*” (medical doctor)

Nurses in the ED also talked about time pressure, working effectively, and not having much time to ask the patients about their preferences. They mentioned that they sometimes tell patients to talk to the doctor after transfer to the ward instead. Time pressure was a common issue:

“*One has to prioritize, if you spend much time on one of the older patients, then there is less time for the other patients in the ED.*” (intern)

Several participants said that older patients were prioritized during hospital admission, but shortages of staff and beds in the ED and on hospital wards can cause long waits for medical examinations. An optimistic nurse in the ED said that the ideal situation would be no waits in the triage part of the ED admission process.

### Healthcare professionals’ attitude towards exploring older patients’ experiences

The attitude and understanding of the healthcare professionals towards older patients’ participation varied. Several participants emphasized that they tried to explore older patients’ experiences, ask about their health problem, provide explanations, and respect their wishes.

An ambulance worker stated that one of his intentions during the journey to the hospital was to give patients an optimal experience and to help them feel respected and cared for. One nurse said that it was important to be professional and provide patient-centered care. This might include actions as simple as welcoming patients with a smile. Several of the medical doctors said they asked their patients about their experiences and views of their medical problem.

One medical medical doctor preferred to sit at the bedside, to improve the quality of the interaction. He said:

“*The patients say what they want if you sit down and ask them.*”

Some doctors and nurses also said that they tried to involve the patients in treatment and care by asking about their health challenges, but found that some older patients did not understand. One medical doctor emphasized the importance of patience in communicating with older patients.

Despite many positive statements, not all the participants could see how to involve older patients. One nurse felt that patient participation was not relevant in the ED. She was not familiar with the concept and held that the decision to admit the patient was made by the doctor in the municipality. An intern said that involvement depends on the patients and whether they are capable of making decisions.

Some nurses and doctors were concerned that there was a shortage of patient participation, with a medical nurse saying:

“*I think it is important that the patient feel he has a right to decide himself. It is good for the patient to be seen and heard and [to feel] that we do not just overrule him by our procedures, which we easily can.*”

There were differences in views on involvement in decision-making about medical treatment. One nurse said that medical treatment is decided by the doctor and is often conducted without asking and involving the patient in the decision-making. A medical doctor pointed out, however, that the final decision about whether to treat is made by the patient, who must be informed of the alternatives.

Several participants focused on avoiding unnecessary hospital admission of older patients. One nurse doubted whether hospital admission was the best alternative for older patients, saying:

“*Older people want to stay at home as long as possible if they know help will come when needed.*”

The study participants emphasized adjustment for end-of-life care, and letting older patients stay at home for as long as possible, and decide for themselves whether they should be admitted to the hospital. One ambulance worker stressed that communication between healthcare professionals, and proper documentation of patients’ functions, statements, and wishes are important to avoid unnecessary transfers.

### Presence of a supportive and demanding next of kin

The interviewees considered patients’ next of kin to be a good source of support when older patients were admitted to the hospital. One ambulance worker commented:

*“Older people often call the next of kin instead of the doctor or the emergency services.”*

He had found that family members were often present when the ambulance arrived at the patient’s home, and provided support with information and practical tasks. According to both doctors and nurses, having the next of kin present during hospital admission is valuable, in particular when providing information to older patients. The interview participants also considered the next of kin as a valuable information source. They know the patients well, can remember better what has been said, and are listened to by healthcare professionals.

Several of the interview participants considered next of kin to be a practical support for older patients. One nurse commented that the presence of someone familiar made older patients feel safe.

“*For a nurse, it is good to know that the patient is not alone in the room, he has his family present, especially when I am busy with other patients. Then I know that next of kin are staying with their loved one, and I ask them to tell me when they are leaving.*” (nurse)

Although next of kin were a valuable source of support and help for healthcare professionals, the nurse emphasized that responsibility for care in the ED lay with the professionals.

Clinicians had several opinions about next of kin. Two nurses felt that patients were taken more seriously if their next of kin was present at admission. According to some of the interview participants, however, next of kin could sometimes be a challenge as their opinions and proposals might not be consistent with the patient’s needs or wishes. Both an ambulance worker and a medical doctor emphasized that patients’ needs and preferences take priority, although the views of next of kin were important.

## Discussion

Our study has identified factors influencing patient participation by exploring healthcare professionals’ views on patient participation during the hospital admission of older patients through the ED. Results indicate that the participation of older patients in the hospital admission process is influenced by five factors.

During hospital admission, routine treatment like assessing the patient’s vital functions is the first priority for clinicians, and they use a medical triage system to prioritize patients who need emergency care [34]. Meeting patients’ physical needs is vital and healthcare professionals need to have good clinical skills to ensure that patients feel safe and receive the right care and treatment in the ED [35]. In this study, the majority of physicians in the ED were interns. This is a challenge and can lead to procedure- and symptom-oriented care [22], with limited involvement of patients in their treatment and care. Andersson et al. [35] reported that medical competencies were valued more than caring competencies in everyday work in the ED, but clinicians in that study agreed that caring competencies were necessary to build a relationship with the patients. Our results support the idea that both medical and caring competencies are important in hospital admission to ensure the involvement of older patients [36].

Participants in the study reported that older patients’ physical frailty and complex health condition may present a challenge to patient participation. The characteristics of older patients reported in earlier studies included being patient, tolerating pain well, hesitating to ask questions, and never complaining [8, 37]. Older patients might therefore become passive recipients of treatment and care, which is typical in the initial stage of illness [38]. Interview participants in our study said that wearing standard hospital clothing and staying in bed may also decrease patients’ willingness to report pain or explain their preferences for treatment and care. There is a risk of less awareness among healthcare professionals of older patients’ needs and preferences, as this group is perhaps not seen as capable of participating in their own care [38]. Older patients might end up being triaged as having more straightforward care needs than is actually the case, because they do not like to ask questions or complain [8].

Availability of hospital resources such as staff and beds influence patient participation in hospital admission, and were reasons given by clinicians in this study for not involving older patients. In a study by Storm et al. [15], some older patients in the ED waited between 3 and 7 h before being admitted to a hospital ward because of a crowded ED. The results in our study also suggest that healthcare professionals seem to prioritize aspects of work other than involving older patients in their treatment and care. The registration of ED patients is time-consuming but it is important to record vital patient information [39]. Research has identified several strategies for handling overcrowding, lack of care efficiency and provision of high-quality emergency care in the ED [8, 14, 15, 36, 39, 40]. Eitel et al. [39] suggested that the emergency severity index triage system could help to prioritize patient needs. A protected time plan for clinicians also can help them plan for changes in patient flow in the ED [15, 39]. The use of nursing care plans in the ED can contribute to increased nurse/patient contact and improve communication between patients and nurses [38].

The interviewees all seemed to have high expectations of themselves and aimed to give high-quality treatment and care during the hospital admission process. The clinicians talked about taking the time to listen to patients’ stories and to talk with them and their next of kin. Allowing patients to provide information about their health challenges, and giving them information about their treatment and care are necessary to involve them in decision-making and for truly informed choice [41, 42]. This is a fundamental value of patient-centered care [1, 42]. Older people want to be informed, heard, and involved in transitional care [8, 15, 21]. Storm et al. [15] found that older patients were dissatisfied with the long wait time for hospital admission and wanted to participate in decision-making about their level of care. A study by Dyrstad et al. [8] showed that patients and family members were not particularly involved in decisions about medical treatment and care during hospital admission. This contrasts with the intentions of clinicians in our study, who aimed to inform patients and involve them in decisions. This might be because of a lack of either time or established routines that involve patients in their treatment and care.

In increasing older patients’ participation, we have to consider whether we want genuine participation, or merely to inform patients about decisions already made by healthcare professionals [43]. Many clinicians in our study seemed to want to provide patient-centered care, respecting patients and taking the time to listen to them. Berwick [42] referred to a patient statement: “They give me exactly the help I need and want, exactly when and how I need and want it” (p. 558), which seemed to be the general ambition in our study. We found that clinicians perceived that older patients could be overwhelmed by the hospital on arrival. They therefore made decisions based on what they perceived as the patients’ best interests. Clinicians should focus on older patients’ views and resources, rather than their frailty, and identify those who are capable of explaining their health challenges and participating in decisions about their treatment and care [44]. Taking a few moments to ask for patients’ stories can be enough to identify their preferences and views [22, 23, 45].

Next of kin were described by the interview participants as fulfilling several roles, including receiving and providing information, and helping the patient to feel safe. This has also been reported in other studies, which found next of kin were important in articulating older patients’ needs and supporting their participation by advocating on their behalf [8, 15, 46]. In our study, next of kin were also perceived as demanding by healthcare professionals. These individuals often advocate for both themselves and the patient. Several of the participants in our study noted that it was important to take patients’ wishes and needs into consideration before those of their next of kin.

We suggest it is important to increase healthcare professionals’ knowledge of the factors influencing the participation of older patients in hospital admission, educate staff to handle complex situations, and facilitate continuity of treatment and care. Dyrstad et al. [8] reported that healthcare professionals need better awareness and knowledge of how to support older patients’ participation. Measures that focus on information and participation of older patients in forthcoming transitions would be helpful. Training to improve provider–patient communication, especially sharing information with patients and their families, talking to patients, and involving them in care planning, would also be useful. Other useful measures include standardizing routines for information exchange, organizing meetings with next of kin to plan follow-up care, and encouraging the next of kin to stay with patients during hospital admission [15].

## Conclusion

This study explored healthcare professionals’ views on patient participation in the hospital admission of older patients through the ED. We found that patient participation is influenced by five factors: routine treatment and care in hospital admission, the patients themselves, availability of hospital resources, especially staff and beds, the healthcare professionals’ attitude towards exploring older patients’ experiences, and the presence of a supportive and demanding next of kin. Some of the participants wanted to involve patients and emphasized that they kept patients constantly informed during hospital admission. A crowded ED ward, time pressure, lack of resources, and procedure-driven care, however, adversely affect the involvement of patients in their treatment and care. Next of kin were considered important in helping older patients to feel safe during hospital admission.

To integrate patient participation as an important element in healthcare, participants suggested that inter-professional meetings and educational programs would be helpful.
